# Influence of Climate Change on Productivity of American White Pelicans, *Pelecanus erythrorhynchos*

**DOI:** 10.1371/journal.pone.0083430

**Published:** 2014-01-08

**Authors:** Marsha A. Sovada, Lawrence D. Igl, Pamela J. Pietz, Alisa J. Bartos

**Affiliations:** United States Geological Survey, Northern Prairie Wildlife Research Center, Jamestown, North Dakota, United States of America; University of Sydney, Australia

## Abstract

In the past decade, severe weather and West Nile virus were major causes of chick mortality at American white pelican (*Pelecanus erythrorhynchos*) colonies in the northern plains of North America. At one of these colonies, Chase Lake National Wildlife Refuge in North Dakota, spring arrival by pelicans has advanced approximately 16 days over a period of 44 years (1965–2008). We examined phenology patterns of pelicans and timing of inclement weather through the 44-year period, and evaluated the consequence of earlier breeding relative to weather-related chick mortality. We found severe weather patterns to be random through time, rather than concurrently shifting with the advanced arrival of pelicans. In recent years, if nest initiations had followed the phenology patterns of 1965 (i.e., nesting initiated 16 days later), fewer chicks likely would have died from weather-related causes. That is, there would be fewer chicks exposed to severe weather during a vulnerable transition period that occurs between the stage when chicks are being brooded by adults and the stage when chicks from multiple nests become part of a thermally protective crèche.

## Introduction

Climate change can have direct or indirect effects on a species and its habitats, which presents unforeseen challenges to conservation. For many species, climate change has led to shifts in phenology, but the mechanisms driving the shifts are poorly understood [1], [2]. There is considerable evidence that spring migration and the timing of breeding for many bird species are occurring progressively earlier than in the past, which is consistent with expected responses to climate change [3], [4]. Species that arrive or breed too early may suffer increasing levels of ecological mismatches to abiotic factors (e.g., encounter adverse weather conditions) or mismatches to the life cycle of other species (e.g., experience inadequate food supplies) and ultimately experience negative consequences to their fitness [5], [6].

The breeding behavior of adult American white pelicans (*Pelecanus erythrorhynchos*; hereafter pelicans) and the physiology of their chicks make the chicks exceptionally vulnerable to unfavorable weather [7], [8], [9]. At hatch, pelican chicks are altricial, naked, and ectothermic; thermal maintenance at this stage of development is aided by nearly continuous parental brooding [8]. During the first 2–3 weeks after hatch, shivering and endothermy develop along with increasing body size and growth of insulative down feathers [10]. Adult pelicans begin to terminate brooding when the chicks are about 2 weeks of age, enabling both parents to leave the colony for extended foraging trips, typically 24 hours [7], [11]. At 14–25 days, the chicks' ability to thermoregulate is not adequately developed to fully protect them from cold weather [11], [12]. To compensate for the loss of thermal protection provided by the parents, chicks from multiple nests form crèches that function as a substitute for continuous parental care [10], [12], especially when ambient temperatures are low (e.g., night, inclement weather) [8], [10], [13], [14]. In a crèche, the chicks are able to retain warmth and protection as long as the crèche is of adequate size [12]; a crèche with too few chicks may fail to function in keeping individual chicks warm, depending on the ambient temperature. There is a transition period between the stage when chicks are being brooded by parents and when chicks form crèches of adequate size to provide thermal protection. During this transition period, chicks are particularly vulnerable to adverse weather conditions [9].

Given that one-half of the entire breeding population of pelicans nest at fewer than 10 colonies in the northern plains [15], maintaining productivity at these northern plains colonies is crucial to the health of the species. At pelican colonies in the northern plains, siblicide, West Nile virus, and exposure to inclement weather (extreme cold and wet, hereafter severe weather) currently are the major causes of chick mortality [7], [9]. Siblicide usually occurs within the first two weeks after hatch [7], when chicks are being brooded and protected from severe weather by adults. Chick mortality caused by West Nile virus does not occur until late in the breeding season (i.e., beginning in mid-July), well after the peak period of chick vulnerability to severe weather [7], [9]. At northern plains colonies in recent years, the only other factor contributing to large numbers of chick deaths has been severe weather [7], [9].

There is an emerging need to better understand the interrelationships among climate change, advanced arrival and breeding of migratory birds, and reproductive output in bird populations [16]. If pelicans are arriving on the breeding grounds earlier and initiating nesting earlier than in the past, we surmised that the number of pelican chicks dying from exposure to severe weather might have worsened. In the northern plains, early spring storms that are characterized by prolonged cold, wet periods are typical, so chicks reaching the transition period earlier in the spring are more likely to be impacted by severe weather than chicks reaching the transition period later. For one of the largest American white pelican colonies in North America, we (1) quantified the advance in the date that pelicans arrive on their breeding grounds, (2) compared the timing of severe weather in recent years to that four decades earlier, and (3) assess the influence of advanced arrival of breeding adults on weather-related mortality of their chicks.

## Study Area and Methods

### Ethics Statement

All collections and handling of pelican chicks were conducted under federal (U.S. Fish and Wildlife Service: MB673019-0, SUP62515-06-0011; U.S. Geological Survey: FBBP20873) and state (North Dakota Game and Fish: GNF02359706) permits and were approved by Northern Prairie Wildlife Research Center's Animal Care and Use Committee (Project Number: NN00.0LLX3). Any use of trade, product, or firm names is for descriptive purposes only and does not imply endorsement by the Government.

### Study Area

Chase Lake National Wildlife Refuge is 19 km northwest of Medina in western Stutsman County, North Dakota (47°01′N, 99°27′W). The area was designated as a National Wildlife Refuge (NWR) in 1908 to protect a persecuted and declining colony of nesting American white pelicans on Chase Lake [17]. The 1,775-ha refuge occurs within the Missouri Coteau physiographic region [18], which is characterized by morainic gently rolling plains interspersed with closed wetlands, prairie pastures, planted grasslands, hayfields, and cropland. An additional 2,474 ha of federal- and state-owned grasslands and wetlands are adjacent to the refuge. Chase Lake is a shallow, 1,150-ha (2008 size estimate) alkaline lake that has no outlet and supports no aquatic vertebrates in most years. During the field study (2004–2008), pelicans nested on 1–3 islands on Chase Lake, and in 2004 only, pelicans also nested on a peninsula (see Sovada et al. [17] for additional details). During those five years, the average number of nests initiated annually was about 13,200. The climate of the region is continental, with cold winters and hot summers, and peak rainfall occurs during the early to mid-growing season. Weather during the growing season is variable and often extreme, with periodic droughts, occasional hailstorms, great fluctuations in temperature, and frequent strong winds.

### First Arrival Dates

Since 1965, biologists at Northern Prairie Wildlife Research Center have been recording spring arrival dates of migratory birds in Stutsman County in central North Dakota [19](LDI, unpublished data). Although the exact date of arrival at this remote colony and subsequent first nest initiation is not known in every year, first arrival dates in the region were collected in a similar manner throughout the 44 years. We believe that the date of first arrival within 60 miles of the colony accurately represents the arrival dates at the because (1) in years in which we closely monitored the colony, first observations of pelicans within 60 km coincided with pelicans arriving at the colony, 2) pelicans can fly hundreds of kilometers in a day, 3) typically more than one observer in the region reported seeing pelicans on the first date of observation, and 4) pelicans are large conspicuous birds and easily observed upon arrival. Furthermore, our observations indicate that courtship flights started immediately upon arrival at the colony, quickly followed by nest initiation [7], and nest initiations can be backdated from known hatch dates. Thus, our assumption that the arrival of the pelicans within 60 km of the colony corresponds well with the arrival at the colony is reasonable and appropriate. We calculated the slope of the linear regression of arrival dates against year to assess advances in arrival date over a 44-year period (1965–2008).

### Defining the Transition Period

For both 1965 and 2008, we estimated a 15-day period (hereafter transition period) during which most chicks would be vulnerable to exposure while in transition from being brooded by adults to being part of a thermally protective crèche. To calculate the beginning of the transition periods for 1965 and 2008, we first estimated the adult arrival dates from the regression line (described above) for the period from 1965 to 2008. Based on our observations [7] and phenology reported by Evans and Cash (1985) [14]), we concluded that a large portion of nests are initiated 7–15 days following arrival of adults at the colony. These early nests produce the cohort of pelican chicks that are most likely to be exposed to severe weather. The start of the transition period was estimated to begin 51 days after the first arrival date, and was based on the average number of days required for courtship and nest building (7 days), incubation (30 days), and chick brooding (14 days) [7], [14], [20]. For 1965 and 2008, we designated the subsequent 15 days as the transition period, during which these chicks would be most vulnerable to severe weather.

### Weather Data

Daily weather data (e.g., maximum and minimum temperatures, precipitation) for years 1965–2008 were compiled from the North Dakota Agricultural Weather Network (NDAWN; http://ndawn.ndsu.nodak.edu/). Continuous data were not available from a single weather reporting station for all 44 years of this evaluation. Therefore, we used weather data collected at the three reporting stations nearest to Chase Lake, including Pettibone (1965–1993, 14 km northwest of Chase Lake), Robinson (1994–2002, 30 km northwest), and Tappen (2003–2008, 21 km southwest).

### Weather Criteria for Severe Weather Days

**Figure 1:**
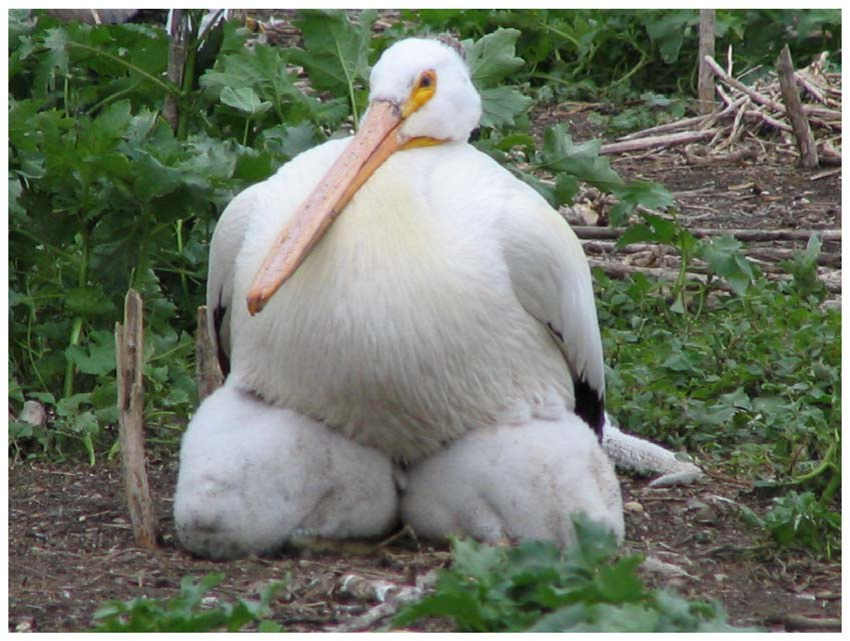
Two older American white pelican chicks (5–6 weeks old) returned to their nest to be brooded during inclement weather (10 June 2006, Chase Lake, North Dakota), but were too large to be covered by the parent. Both chicks later died.

We established criteria to define the weather conditions that put chicks at risk of exposure. Criteria for at-risk days (hereafter severe weather days) included (1) maximum daily temperature of ≤10°C; (2) measurable precipitation and minimum daily temperature of ≤10°C; or (3) minimum temperature ≤10°C and measurable precipitation the prior day. We chose 10°C because, in laboratory experiments, Evans [12] showed a thermal advantage of crèching at 10°C ambient temperature that was not apparent at 20°C and 30°C. Moreover, we observed chicks shivering in response to ambient temperatures that were between 10 and 15°C. Other defining conditions were somewhat arbitrary, but were supported by published accounts [21], [22], [23] and our previous observations that cold and rainy conditions were clearly detrimental to chicks during the transition period. Younger pelican chicks lack the waterproof contour feathers of adults to help maintain their body temperature during exposure to cold and rainy conditions; when downy pelican chicks are wet, their body temperatures can fall as much as 0.5°C per minute [21]. When wind, precipitation, and low temperatures combined to make conditions particularly severe, we observed older chicks suffering as well (e.g., shivering, returning to nests to be brooded despite being too large to fit under an adult; Fig. 1).

### Comparing Severe Weather Days in the 1965- and 2008-based Transition Periods

For the 2004–2008 weather data, we compared the number of severe weather days that occurred during the 1965- and 2008-based transition periods (paired t-test; PROC TTEST, [24]). We also used linear regression (PROC REG, [24]) to evaluate whether the number of severe weather changed between 1965 and 2008 during either the 1965- or 2008-based transition periods.

### Direct Surveillance

Each year (2004–2008) during the early part of the breeding season, we assessed nesting activities at the colony with spotting scopes from mainland vantage points. We did not access islands during sensitive periods of courtship and nest initiations to avoid disturbing the birds and for safety reasons (typically ice is not off the lake when pelicans begin breeding activities). Once access to the islands was reasonable, we monitored nesting activities at least once per week, but typically three or more times per week, from vantage points that were located on the islands such that disturbance to nesting birds was minimal. Strong winds are prevalent in this region, and safe access to the nesting islands often was not possible on a daily basis, especially during or immediately following a storm. During visits to the colony, we collected information on nesting activity and noted if there were unusual numbers of dead or moribund chicks, indicating a mortality event (potentially caused by weather, disease, or predation) had occurred or was in progress. Generally, deaths, injuries, or illness caused by weather, disease, siblicide, or predation are distinguishable based on evidence or symptoms of sick or dying birds. We collected information on the environmental conditions (e.g., temperature, precipitation, evidence of hail or strong winds), the number of sick or dying chicks, the behavior and symptoms of sick chicks, and other evidence (e.g., external trauma) to help identify the cause(s) of mortality or waning health (see Sovada et al. [9]). If evidence pointed to disease (e.g., ataxia, paralysis, tilted head, lethargy, convulsions, disorientation), or there was no obvious cause of death, a sample of chicks was submitted to the U.S. Geological Survey's National Wildlife Health Center in Madison, Wisconsin, for diagnostic tests.

## Results

### Arrival Dates and Transition Periods

**Figure 2:**
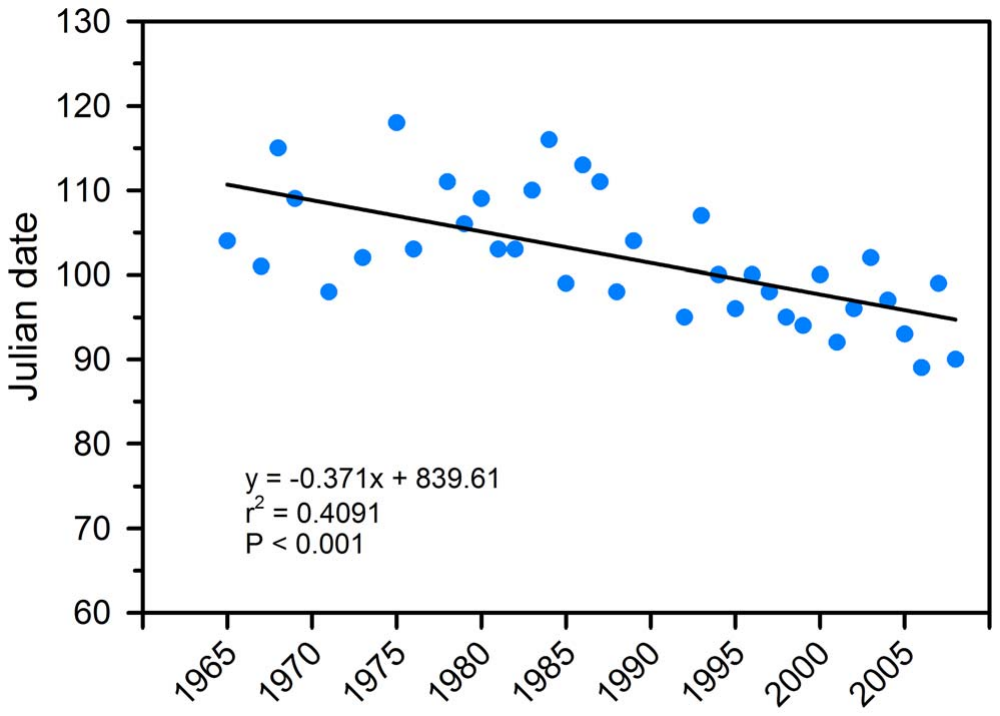
The date that American white pelicans first arrived each year at a breeding colony in Chase Lake National Wildlife Refuge in Stutsman County, North Dakota, during a 44-year period (1965–2008). Pelicans showed a trend toward earlier arrival, with arrival dates advancing approximately 16 days in the 44-year period. Arrival dates were not recorded for 1966, 1970, 1972, 1974, 1977, 1990, and 1991.

First arrival dates for pelicans within 60 km of Chase Lake NWR were recorded for 37 of the 44 years (1965–2008). Pelicans showed a trend (r^2^ = 0.41, P<0.0001) toward earlier arrival between 1965 and 2008, with arrival dates advancing approximately 16 days (Fig. 2; 21 April in 1965 vs. 5 April in 2008). Similar to pelican breeding behavior reported by other studies at northern colonies [14], [25] and earlier studies at Chase Lake [26], we documented pelicans commencing courtship and nesting activities immediately upon arrival at the colony site during our field study (2004–2008). Therefore, it is reasonable to assume that the interval between arrival and nest initiation was similar over the 44 years, and that the transition period when chicks are most vulnerable to mortality from exposure also likely advanced by about 16 days. Transition periods were estimated as 11–25 June based on 1965 phenology and 26 May–9 June based on 2008 phenology. Although we observed nest initiations extend over a 3 month period, the majority occurred early in the breeding season and this large cohort from early nests was the most likely to be exposed to severe weather.

### Severe Weather Days (5-year Field Study Period and Over 44 Years)

**Figure 3:**
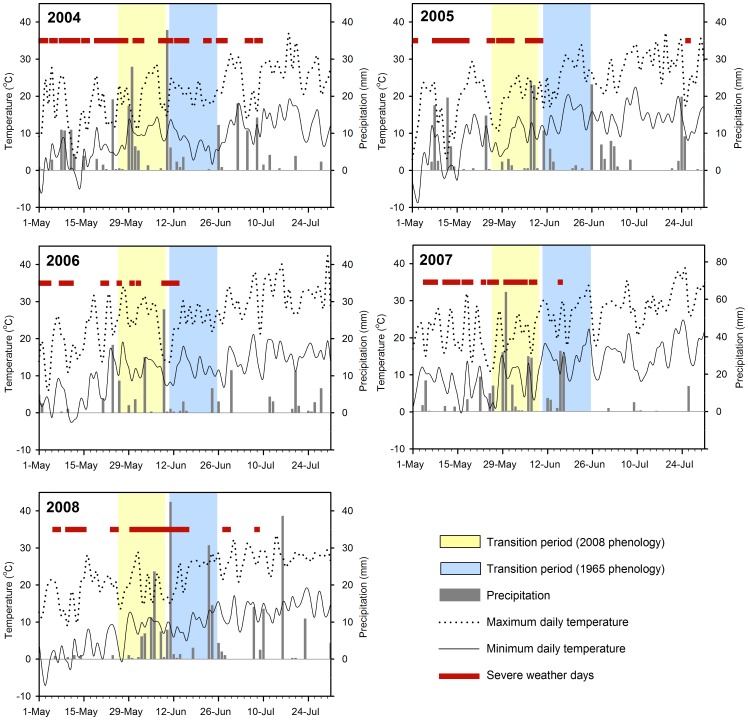
Daily precipitation, maximum and minimum daily temperatures, and severe weather days during May through July in 2004–2008 at Chase Lake, North Dakota. Also indicated are the transition periods (time between chicks being brooded by adults and when chicks become part of a thermally protective crèche) for both 1965 and 2008 breeding phenologies of American white pelicans. Note scale difference in precipitation 2007.

During the five years (2004–2008) of our field study, pelican chicks were more likely to be exposed to severe weather conditions than they would have been if adults had followed a 1965-based phenology pattern (t_4_ = 2.68, P = 0.055; see Fig. 3). During 2004–2008, on average, there were 9.0 (SD = 1.5) severe weather days in the 2008-based transition period and 3.6 (SD = 1.4) severe days in the 1965-based transition period.

Over the 44-year period, severe weather days were more frequent during the 2008-based transition period than the 1965-based transition period (t_43_ = 3.25, P = 0.002). The number of severe weather days across the years, however, did not exhibit evidence of a linear trend within each transition period for either the early (r^2^ = 0.05, P = 0.13) or the late (r^2^ = 0.01, P = 0.49) arrival scenarios. Thus, we have no evidence to support any change across the 44 years in the frequency that severe weather occurred or in the timing of severe weather in the spring and early summer.

### Observed Mortalities Attributed to Severe Weather, 2004–2008

In the five years (2004–2008) of field study, we observed extraordinary weather-related mortality of chicks on several occasions, underscoring the devastating effects of cold and rainy conditions on chick survival at the Chase Lake colony. The chicks that died during weather events were mostly in the transition period between being brooded and being part of a protective crèche. The following annual narratives describe notable losses attributed to severe weather events and periods of inclement weather. Losses to other factors (e.g., West Nile virus, siblicide) during 2004–2007 are described in Sovada et al. 2008, 2013 [7], [9].

In 2004, on 27 May, we observed normal activities and behaviors at an island subcolony that included approximately 6,000–8,000 pelican nests (Table 1). Most adults were attending chicks that were about 2–3 weeks old. In late May to early June, the entire region experienced an extended period of cold (low temperatures: mean = 5.6°C, range = 1.1–13.3°C), wet (precipitation, 78 mm), and windy (sustained maximum ∼40 km/hr with gusts to 65 km/hr) weather (Fig. 3, 2004). We were able to access the island on 13 June, but by that date, only a few live chicks (<50) remained and nearly all the nesting adults had deserted the subcolony. Many chick carcasses were present, but many others likely had decomposed or were scavenged by ring-billed gulls (*Larus delawarensis*) or California gulls (*L. californicus*), which also nest in large numbers at Chase Lake. Based on remaining carcasses, we estimated that at least 2,000 pelican chicks perished as a result of the adverse weather. Chick deaths at the Medicine Lake pelican colony in northeastern Montana were also attributed to this regional weather event [9].

**Table 1:** American white pelican first arrival dates, nest initiation dates, and the numbers of nests and chicks for individual subcolonies at Chase Lake, North Dakota, 2004–2008.

Year	Date of first arrival	Number of nests at subcolonies				Total nests	No. of chicks^2^
		North island	South island	Mainland peninsula	Middle island		
2004^1^	6 Apr	7,000	200	7,000	500	16,500	0
2005	3 Apr	1,765	7,387	0	179	9,331	<300
2006	31 Mar	3,202	14,100	0	0	17,302	11,020
2007	12 Apr	10,105	1,157	0	0	11,262	531
2008	3 Apr	2,716	8,825	0	0	11,541	86

^1^ Nest numbers in 2004 are estimates based on incomplete data; adult pelicans abandoned the nests prior to the scheduled survey.

^2^ The surveys of chicks were conducted on the following dates: 15 August 2005, 14 July 2006, 28 June 2007, 12 August 2008. No pelican chicks survived in 2004.

In 2005, there were 9,331 pelican nests on three islands at the Chase Lake colony (Table 1). On 16 June 2005, we observed that nearly one-half of approximately 1,600 chicks on one island subcolony were dead. This subcolony had the earliest nest initiations (approximately 1,700 nests) of the three island subcolonies, and most of the dead chicks were about 2–4 weeks old. The carcasses were not fresh enough to necropsy, but cool (minimum temperatures were around 10°C), wet, and windy weather during the previous week likely contributed to these deaths (Fig. 3, 2005). Weather prevented our access to the islands during this period. A catastrophic weather event during the night of 2–3 July (sustained winds of 80–97 km/hr) likely caused additional deaths of about 1,500 chicks, which we recorded on 5 July. After this event, we found dead chicks on all islands, of all ages, often in piles, and already too decomposed to necropsy.

In 2006, the first pelicans arrived in the area on 31 March, the earliest arrival reported for the colony between 1965 and 2008 (Table 1). In that year, over 17,000 nests were initiated on two islands at Chase Lake. Weather in 2006 was relatively mild compared to the previous two breeding seasons, with only five days (9–13 June) categorized as severe under our criteria (Fig. 3, 2006). On 11 June, we recorded approximately 400 dead chicks, about 2–3 weeks old, which presumably succumbed to the cold, wet, and windy weather. We observed no other obvious weather-related losses at the colony during the remainder of the breeding season. From aerial photographs taken on 13–14 July, we counted approximately 11,020 chicks on the two islands. This represented 78% nest success (assuming one surviving chick per nest) at one island subcolony and 60% success at the other. In this year of relatively few weather-related deaths, the levels of productivity were comparable to or higher than the productivity (0.34–0.68 young per nest) reported at Chase Lake in the early 1980s [27].

In 2007, the Chase Lake colony included 11,262 pelican nests on two islands (Table 1). The colony once again suffered nearly complete failure in productivity, in part because of severe weather. Most weather-related losses resulted from persistent cold and rainy conditions rather than from a specific storm event (Fig. 3, 2007). From 1 May to 18 June, daily minimum temperatures averaged 8°C (range −1–14°C), and the area received 322 mm of precipitation, of which 249 mm came during a 3-week period from 29 May to 18 June. Average rainfall for this period is <76 mm. A storm on 7 June included hail. Many nests hatched around 19 May, making many chicks vulnerable to severe weather during late May and particularly in early June. By 5 June, the effects of cold, wet, and windy weather were evident. We observed dead chicks in nests on the east side of the north island (nests hatch around 19 May). Nearby, we were video monitoring nine nests that hatched during 18–23 May. Among the nine nests, six had failed and their loss was attributed to poor parental care (low exchange rate between parents, followed by low chick feeding rates), which likely was related to poor weather conditions. Unusually high mortality of young chicks continued through June. On 28 June, we estimated that there were only 531 surviving chicks on the south island and <50 chicks on the north island. The older age of these chicks likely contributed to their survival during the period of severe weather.

In 2008, we estimated that there were 11,541 pelican nests on two islands in Chase Lake. Prolonged cold and wet weather during the last week of May through the second week of June (Fig. 3, 2008) again impacted chick survival. Nearly 130 mm of precipitation fell over 15 days during that 3-week period. The average daily minimum (8°C) and maximum (19°C) temperatures were 5°C and 8°C lower than the respective averages during the same period in 2006 (a productive year for pelicans at this colony). We visited the colony on 10 June 2008 and observed that a large portion (about 80%) of the approximately 1,700 chicks that were between 2 and 3 weeks old were dead. Many of the surviving, apparently healthy chicks in this cohort were observed shivering at ambient temperatures of >10°C. At areas on the islands that were most impacted by this severe weather, we counted five dead chicks for every live chick. The dead chicks appeared to be in excellent body condition just prior to death, based on their healthy levels of fat reserves and low ectoparasite (e.g., pouch lice [*Piagetiella peralis*]) burdens. Some of the dead chicks were found in small groups of 3–4 chicks, but most of the dead chicks were associated with a nest bowl. We did not test the carcasses for disease, because evidence strongly suggested that the deaths were related to weather. Moreover, the nests with eggs or with chicks younger than 2 weeks old that were attended by an adult appeared to be unaffected by this period of severe weather. The weather remained unfavorable through the rest of June, with another 40 mm of rain and average daily low temperatures of 11°C. There continued to be a gradual loss of chicks during the remainder of June, largely attributed to the persistent cold, wet weather conditions.

## Discussion

American white pelicans breeding at the Chase Lake colony have advanced their spring migration and onset of breeding by over two weeks in four decades. The advance in arrival to breeding areas by pelicans that we describe is similar to what has been documented for a growing number of species in this region [3] and throughout the world [1], [28]. Earlier arrival of pelicans at Chase Lake has led to earlier nesting, and chicks from these nests are now exposed to more days of severe weather during their most vulnerable period—the transition from being brooded by parents to forming thermally protective crèches with other chicks. During four of our five years of field study at the Chase Lake colony, the apparent consequence of advanced arrival on the breeding grounds by pelicans was poor breeding success, largely related to chick deaths caused by inclement weather. Although spring temperatures in the northern plains of North America have increased [29], [30], the timing of severe weather in the Chase Lake area has not changed. Thus, the conditions now experienced by the pelican chicks during the critical transition period between parental brooding and chick crèching have likely worsened and breeding success has suffered.

Crèching is a common survival strategy observed in young of many colonial-nesting bird species. Although the proximate cause of this behavior remains unknown, several functions have been advanced to explain its adaptiveness, including thermal protection, protection from predators, and protection from aggression by unrelated adults [31], [32]. In the American white pelican, Evans [11], [12] showed that crèched chicks save energy and are afforded thermal protection compared to isolated chicks; thus, crèching in this species appears to function—at least in part—as a substitute for constant parental care and brooding for older chicks.

At the largest island in Chase Lake, habitat conditions (description follows) compromised the ability of chicks to form crèches large enough to provide thermal protection, and these conditions may have amplified the number of deaths caused by severe weather. Pelicans first nested on this island in 2002, after a wet period that began in mid-1993 [33] resulted in a dramatic rise in the water level at Chase Lake, inundating the historical nesting islands and created new nesting islands as peninsulas were cut off from the mainland [17]. Pelicans shifted nesting to the newly formed islands. On the largest island, pelicans selected relatively open areas for nesting in each year, but the aggressive growth of some plant species (*Cyclachaena xanthifolia, Urtica dioica, Toxicondendron radicans, Chenopodium leptophyllum*) degraded the quality of habitat for nesting pelicans as the season progressed. Tall, dense vegetation obstructed movements by chicks and, ultimately, impeded the formation of larger crèches. The crèches that we observed on this island often were isolated and small (2–3 chicks). Studies addressing thermoregulation in pelican chicks report the importance of crèches and crèche size for warmth and the survival of chicks [10], [12]; however, what critical mass is needed for adequate thermal protection probably varies with conditions. It is reasonable to assume that a crèche of 2 or 3 chicks does not protect against severe weather. The inability of chicks to form crèches of adequate size likely was particularly detrimental in the years of our field study with persistent cold, wet weather.

The American white pelican is a species with a k-selected life history (i. e., characterized by larger body size, smaller clutches, lower productivity, and longer life spans); as such, it is assumed to be less able to adjust its breeding phenology in response to rapid climate change than are r-selected species [34]. Yet, similar to our findings for pelicans, such phenological adjustments by k-selected and other bird species have been observed, and some of these studies also noted increased hypothermia-induced chick mortality attributed to advanced breeding schedules related to climate change [35], [36]. For example, in Finland, the common buzzard (*Buteo buteo*) breeds 11 days earlier in the spring than it did 30 years ago as a result of warmer spring temperatures [36]. Consequently, buzzard chicks now face a higher risk of hatching into less favorable (i.e., colder and wetter) conditions than they did three decades earlier; rain increases the risk of hypothermia and mortality for buzzard chicks and degrades hunting conditions for adults. Similarly, the black grouse (*Tetrao tetrix*) in Finland has advanced egg laying and hatching in response to warmer spring temperatures, but because summer temperatures and the onset of ideal hatching conditions have not advanced, grouse chicks are now experiencing colder post-hatching conditions, hypothermia, and increased risk of predation [35]. These examples illustrate the consequences of phenological shifts in response to climate, with detrimental impacts on productivity.

Pelicans are opportunistic foragers, and their common prey (salamanders, crayfish, minnows, rough fish) typically are accessible and relatively abundant in prairie pothole wetlands throughout the breeding season (e.g., Deutschman and Peterka [37]). However, prey availability can be impacted by overwinter survival [37], wetland conditions [38], and other factors in the region. Our field study was conducted during a long period of relatively wet conditions [33], and the wetland basins in the region contained ample water and presumably adequate but unpredictable prey populations [39], [40]. Although we did not measure food availability, we also did not observe any direct evidence indicating that food resources were compromised (e.g., extensive winter kill of fish in the region, major switch in conventional diet in regurgitate of adults or chicks) during the years of our field study. Yet, we cannot discount the possibility that climate change might impair current or future food availability and foraging efficiency (e.g., increased travel distance, reduced feeding rates, degraded food quantity or quality) and ultimately breeding success [38]. Indeed, water depth, areal extent of wetlands, wet-dry cycles, and vegetation patterns within prairie wetlands are driven by climate [41]. Poiani and Johnson [41], [42] developed a climate-based simulation model of a typical semipermanent prairie wetland to evaluate the amount and distribution of emergent cover and open water in time and space under a climatic-warming scenario. The model predicted substantial increases in overall emergent vegetative cover and declines in open water due to warming, and the wetland was completely choked by emergent vegetation by the fourth year of the simulation. In this region, pelicans typically forage in shallow open areas of wetlands [7], [20]. Because pelicans dip their bill in the water and scoop to gather food, they tend to avoid areas with extensive cover of emergent vegetation. Moreover, earlier drying of wetlands as a result of climate change will exert selective pressure toward more rapid metamorphosis of larval tiger salamanders (*Ambystoma tigrinum*), a primary prey item of American white pelicans in this region, and thus limit the size of the larvae and the amount of time they are available to pelicans [38]. Johnson [43] suggested that pelicans at this colony might experience high rates of nest abandonment during periods of low water levels because adults must travel further and spend more time foraging.

The northern plains are renowned for annual variability in weather [44], [45]. Even without an advance in pelican arrival to the Chase Lake colony and the resulting advance in breeding phenology, it is likely that in some years significant numbers of pelican chicks perished during severe weather [46]. Nonetheless, species in this region presumably have evolved mechanisms to cope with weather-related phenomena, such as drought, storms, and temperature extremes [47], and populations of long-lived species, such as the American white pelican, ostensibly can withstand occasional years of catastrophic reproductive failure (e.g., Diem and Pugesek [22]). However, the earlier timing of pelican arrival and breeding at Chase Lake has likely resulted in more frequent and severe losses of pelican chicks than might have occurred if arrival and breeding on the colonies had not advanced during the past four decades. The nearly complete reproductive failure of the Chase Lake colony in four of the five years of this evaluation suggests that pelicans may be experiencing population-level effects attributable to climate change and raises concerns about the ecological consequences (population instability, decline, extirpation) of projected changes in the climate in future decades.
